# Whole exome sequencing identifies novel variants of PIK3CA and validation of hotspot mutation by droplet digital PCR in breast cancer among Indian population

**DOI:** 10.1186/s12935-023-03075-6

**Published:** 2023-10-11

**Authors:** Rahul Kumar, Rakesh Kumar, Harsh Goel, Sonu Kumar, Somorjit Singh Ningombam, Imran Haider, Usha Agrawal, Svs Deo, Ajay Gogia, Atul Batra, Ashok Sharma, Sandeep Mathur, Amar Ranjan, Anita Chopra, Showket Hussain, Pranay Tanwar

**Affiliations:** 1 Dr B. R. A.-Institute Rotary Cancer Hospital, All India Institute of Medical Sciences, New Delhi, India; 2 Department of Gastroenterology & HNU, All India Institute of Medical Sciences, New Delhi, India; 3 National Institute of Pathology, New Delhi, India; 4 Department of Biochemistry, All India Institute of Medical Sciences, New Delhi, India; 5 Department of Pathology, All India Institute of Medical Sciences, New Delhi, India; 6 Division of Molecular Oncology, National Institute of Cancer Prevention and Research, Noida, India

**Keywords:** Breast cancer, Exome sequencing, PIK3CA, ddPCR, MD simulation

## Abstract

**Background:**

Breast cancer (BC) is the most common malignancy with very high incidence and relatively high mortality in women. The PIK3CA gene plays a pivotal role in the pathogenicity of breast cancer. Despite this, the mutational status of all exons except exons 9 and 20 still remains unknown.

**Methods:**

This study uses the whole exome sequencing (WES) based approach to identify somatic PIK3CA mutations in Indian BC cohorts. The resultant hotspot mutations were validated by droplet digital PCR (ddPCR). Further, molecular dynamics (MD) simulation was applied to elucidate the conformational and functional effects of hotspot position on PIK3CA protein.

**Results:**

In our cohort, PIK3CA showed a 44.4% somatic mutation rate and was among the top mutated genes. The mutations of PIK3CA were confined in Exons 5, 9, 11, 18, and 20, whereas the maximum number of mutations lies within exons 9 and 20. A total of 9 variants were found in our study, of which 2 were novel mutations observed on exons 9 (p.H554L) and 11 (p.S629P). However, H1047R was the hotspot mutation at exon 20 (20%). In tumor tissues, there was a considerable difference between copy number of wild-type and H1047R mutant was detected by ddPCR. Significant structural and conformational changes were observed during MD simulation, induced due to point mutation at H1047R/L position.

**Conclusions:**

The current study provides a comprehensive view of novel as well as reported single nucleotide variants (SNVs) in PIK3CA gene associated with Indian breast cancer cases. The mutation status of H1047R/L could serve as a prognostic value in terms of selecting targeted therapy in BC.

**Supplementary Information:**

The online version contains supplementary material available at 10.1186/s12935-023-03075-6.

## Background

Breast cancer (BC) is one of the most common diagnosed cancer in females, with 11.7% new cases and 6.9% mortality reported worldwide in 2020. While in India, it accounted for more than 178,361 new cases in 2020, with 7.7% of the total incidence and contributed more in terms of BC-related death [1]. BC is heterogeneous in nature which encompasses a distinct group of diseases [2]. It is developed due to genetic as well as epigenetic alterations in the cells of terminal duct lobular unit [3]. Consequently, signalling pathways that regulate the overall fate of cells are dysregulated and lead to an uncontrolled cell proliferation [4]. There are various signalling pathways involved in the pathogenesis of BC, including P53, ERK, PTEN, ATM, and PIK3/AKT regulates cell growth, cell proliferation, cell differentiation, metabolism, and apoptosis, while Wnt/βcatenin governs stem cell renewal, cell proliferation, and differentiation [5–12].

Phosphoinositide 3-kinase (PI3K) is an intracellular pathway that significantly regulates cell growth, migration, metabolism, and survival [13, 14]. PI3K is a heterodimeric lipid kinase enzyme consisting of p85 regulatory subunit and p110 catalytic subunit encoded by PIK3R1/2/3 and PIK3CA/B/D depending upon its various isoforms, respectively [15]. PIK3CA is one of the most common isoforms and is found to be a dysregulated gene in breast cancer [16, 17]. PIK3CA (p110) activates PI3K through its kinase domain, which phosphorylates various downstream signalling proteins. At the same time, this activity is further regulated by the helical domain, which receives an inhibitory signal from regulatory subunit (p85) [18, 19]. Alteration in the catalytic domain leads to constitutively active state of PIK3CA and makes it no longer reliant on upstream stimuli. As a result of prolonged activation, it encourages to escape of apoptosis and induces oncogenic transformation [20, 21].

Recent clinical data mark the importance of mutation profiling of PIK3CA. PIK3CA consists of 20 exons, and each exon encodes a specific domain with its inevitable function [22]. However, only exon-specific mutation profiling has been reported using many conventional techniques. In genomics, the whole exome sequencing (WES) method of massively parallel sequencing offers high throughput to determine nucleotide arrangement within the entire exomes or a specific protein-coding region [23]. In this study, we have performed WES of breast cancer tissues of Indian population and identified top mutated genes. Resultant hotspot mutations of selected gene were further validated with Sanger sequencing and droplet digital PCR (ddPCR). Additionally, the effects of screened mutations on the protein structure and dynamics were examined through structural modelling and simulation studies.

## Methods

### Patient’s cohort

Forty-five primary BC cases were recruited with informed written consent taken from the breast cancer clinics at All India Institute of Medical Sciences (AIIMS), New Delhi, India. The study was conducted in accordance with the declaration of Helsinki, and approved by the Institutional ethics committee of AIIMS New Delhi for a study involving humans. During surgical intervention, the tumor tissue was collected from the core of the tumor in a vial containing 1XPBS (Phosphate buffer saline). About 3–5 mL of peripheral blood was also drawn in EDTA (ethylenediaminetetraacetic acid) vial from the same patients and used as a control for somatic mutation calling. The samples were cryopreserved in liquid nitrogen and stored at -80 °C for further use. All the samples were reviewed for their hormone receptor status, TNM (Tumour node metastasis) stage, and histological classification.

### DNA extraction

Genomics DNA (gDNA) from tumor and blood samples were extracted using QIAamp Fast DNA Tissue/Blood Kit (Qiagen) as per instruction provided by the manufacturer. Elution was done with a final volume of 50 μl followed by DNA quantification through Qubit dsDNA BR Assay kit (Invitrogen). A 0.8% agarose gel electrophoresis was used to check the quality and integrity of genomic DNA.

### Whole exome sequencing

After an initial assessment of DNA, a concentration of 200ng gDNA was used for library preparation. SureSelect^XT^ library prep kit protocol by Agilent was used as per manufacture protocol followed by whole exome capture using AGV5 + UTR probes. Prepared libraries were quantified using Qubit dsDNA HS Assay (Invitrogen™). The obtained libraries were pooled and diluted to final optimal loading concentration before cluster amplification on the Illumina flow cell. Once the cluster generation is completed, the clustered flow cell is loaded on to Illumina HiSeqX instrument to generate 150 bp paired end reads.

### Data processing and variant calling

The quality of sequenced reads were monitored and trimmed to remove low-quality reads as well as primer contamination using FastQC (version 0.11.9) and Trimmomatics (version 0.36) tools, respectively [24, 25]. Trimmed reads were aligned to the human reference genome (GRCh38.p7) using Burrows-Wheeler Aligner (BWA) in the default parameter [26]. Aligned reads were further processed, sorted, and indexed with SAMtools [27]. After that, we mark duplicates with Picard and base quality score recalibration (BQSR) using Genome Analysis Toolkit (version- 4.1.9.0). Reads have mapping quality < 60 were removed, and high-quality reads with genotype quality score (GQ) and depth score ≥ 30 were used to discover somatic variants by Mutect2 [28]. Each variants were supported by > 5 reads and annotated by ANNOVAR with a different database, such as exac03, avsnp150, and dbnsfp42. The resultant variants were visualized through Integrative Genomics Viewer (IGV) web application [29, 30].

### Validation by Sanger sequencing

The forward and reverse primers of exon 20 of PIK3CA gene were designed by the Primer3 tool [31]. The gene was amplified using the specific primer (Table S1), which cover H1047L/R mutations. PCR was performed with 100ng of gDNA, 1 μl of each primer (10 μM/μL), and 12.5 μL of GoTaq® G2 Hot Start Master Mixes (Promega) with a final volume of 25 μL. Amplification was done under the following thermal condition – initial denaturation at 95 °C for 5 min, 40 cycles of denaturation, annealing, and elongation, at 95 °C, 65 °C, and 72 °C for 30 s each, respectively, followed by final elongation at 72 °C for 10 min. The PCR product was sequenced with ABI 3500 Genetic Analyzer (Thermo Fisher Scientific).

### ddPCR workflow

ddPCR is being considered an effective approach to detect mutation with a low copy number. Rare event detection (RED) assay was used to check the known mutations c.3140 A > G (p.H147R) in exon 20 of PIK3CA gene. Details regarding the assay IDs have been provided in Table S2. A reaction mixture of 20 μl was prepared by using ddPCR supermix for probes (No dUTP), 50ng gDNA, 20X primer-probe assay for each wild and mutant type, and nuclease-free water. In order to generate droplets, the cartridge having 20 μl of reaction volume and 70 μl of droplet generation oil in a subsequent lane covered with a gasket was placed into QX200 Droplet Generator. Once the droplets were generated, 40 μl from each sample was transferred into a 96-well plate and sealed with sealer. A sealed plate was used for amplification by using the program: 95˚C for 10 min, followed by 40 cycles of 94˚C for the 30s and 52.4˚C for 1 min, and lastly, 98˚C for 10 min with a temperature increase rate set at 2 ˚C per second. The amplified products were read with a QX200 Droplet reader for detecting wild and mutant droplets, and analysis was done by using QuantaSoft software.

### System preparation and molecular dynamics (MD) simulation

MD simulation was used to study the structural dynamics of both wild (WT) and mutant (MT) type proteins with various missense mutations found in exon 20. The tertiary structure of PIK3CA kinase domain was obtained from RSCB-Protein Data Bank (PDB ID: 4OVU) [32]. The PyMOL was used to remove other domain and non-protein molecules, and the resulting structure was considered as WT. While MTs structures (H1047R, H1047L) were modelled in Modeller10.1 using WT as a template [33]. Structural similarities between WT and MT models were assessed by measuring the root mean square deviations (RMSDs) between them (WT and MTs). In addition, the stereochemical and geometry of modelled MT structures were also examined through inspecting Ramachandran plots and ProSA (Protein Structure Analysis) tool, as discussed previously in our own study [34].

Molecular dynamics simulations of 100ns for all structures were performed using GROMACS 5.0 association with CHARMM force field as described previously [35, 36]. Initially, “pdb2gmx” module was used to create GROMACS-compatible coordinates and topology files, and then each system was solvated using “editconf” module. To achieve systems solvation, TIP3P water model was used, and each protein structure was placed in a triclinic box. A periodic boundary distance of 1.2 nm was maintained, and electroneutrality of the system was accomplished by adding Na^+^ and Cl^−^ ions, followed by energy minimization through the steepest-descent method. After this, two equilibration phases were carried out for simulation by utilizing positional strains as NVT (constant number, volume, and temperature) and NPT (constant number, pressure, and temperature) ensembles for 100 and 500ps at 300 K and 1 bar, respectively. All bonds were constrained using the linear constraint solver (LINCS) algorithm, and long-range electrostatic interactions were met through the PME (Particle Mesh Ewald). The Parrinello-Rahman barostat and V-scale were used to maintain the temperature and pressure of the system, respectively. Finally, a production run of 100ns was performed for WT and MTs systems in triplicates. All MD analyses were done through GROMACS utility modules such as gmx energy, gmx rms, gmx rmsf, gmx, gyrate, gmx sasa, gmx hbond, gmx covar, gmx anaeig, gmx analyze. Secondary structures formation during simulation was calculated through do_dssp module [37].

Essential dynamics was used to obtain the key motions along with first three principal components (PCs) which comprise maximum motions. It will help us to study the biological significant motions related to protein functions [38, 39]. A covariance matrix was created after excluding translational and rotational movement from the trajectories. Diagonalized covariance matrix showed eigenvalues along with corresponding eigenvectors. The stabilized trajectories of MD simulation with cosine values ≤ 0.2 were used for PC analysis and were limited to backbone atoms only [40].

## Results

### Patient characteristics and sequencing statistics

An overview of the clinical and pathological details of the forty-five cases are summarized in Table 1 including age, tumor size, lymph node, distant metastasis, hormonal and Her2neu receptor status, as well as their survival data. All the cases diagnosed with primary BC had a median age of 51. The tumor (T) size of T2 category was predominantly observed (51%), followed by T4 (27%). Approximately 62% of cases had at least one axillary lymph node infiltrated with a tumor. A total of 25/45 cases (55%) were positive for either estrogen, progesterone or both, while 21/45 cases (47%) exhibits amplification of Her2neu receptor. Moreover, the maximum number of cases had HR+/HER2- molecular phenotype followed by HR+/HER2+.

WES data were showed an average of 66.47 million reads per sample with 150 bp as the mean read length. The data was satisfactory for further analysis as more than 99.34% of the reads were aligned to the human reference genome. The missense mutations were found to be more common in analysis, followed by nonsense mutations and frameshift deletion. Among detected mutations, the single nucleotide substitution variants were the commonest, followed by insertion and deletion. Among 6 theoretically possible single nucleotide variants (SNV) classes, C > A was the most common, followed by C > T. PIK3CA was one of our cohort’s top mutated gene, with a somatic mutation rate of about 44.4%.

**Table 1:** Clinical-pathological characteristics of enrolled BC patients

Clinicopathological Parameter (N = 45)	(N = 45)
Median Age (in years)	51
Tumor stage	
T1	3 (7%)
T2	23 (51%)
T3	7 (15%)
T4	12 (27%)
Node status	
N0	17 (38%)
N+	28 (62%)
Hormone receptor (HR) Status	
HR+	25 (55%)
HR-	20 (45%)
Her2 Status	
Her2 +	21 (47%)
Her2 -	24 (53%)
Molecular type	
HR+/HER2-	14 (31%)
HR+/HER2+	11 (25%)
HR-/HER2+	10 (22%)
HR-/HER2-	10 (22%)
Survival status	
Live	39 (87%)
Dead	6 (13%)

### Mutational landscape of PIK3CA gene

**Fig. 1:**
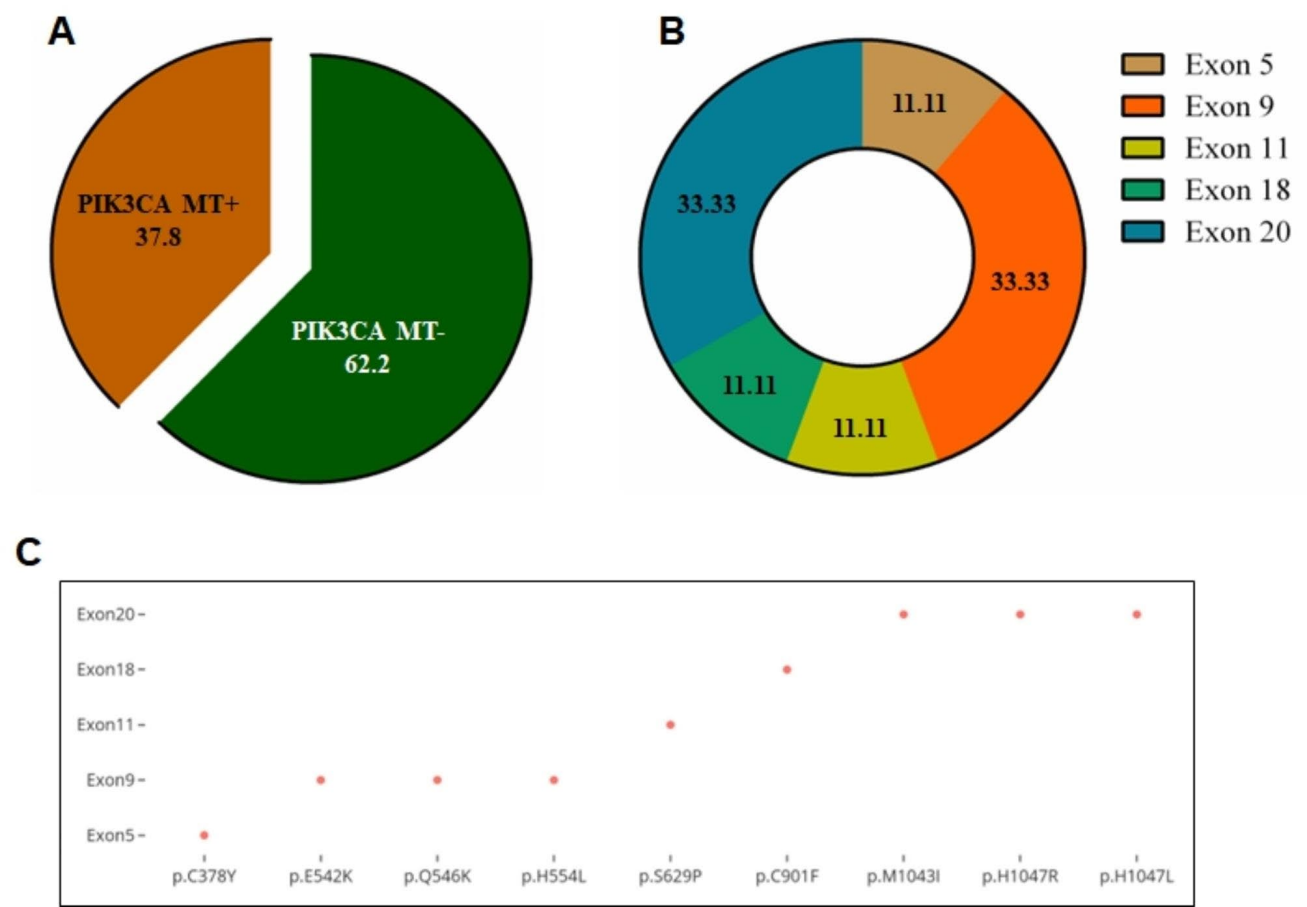
Mutation profiling of PIK3CA gene in breast cancer. (**A**) Alteration of PIK3CA mutation observed in enrolled patients. (**B**) Frequency of mutation throughout the coding region. (**C**) Distribution of point mutation over the entire exon region

**Fig. 2:**
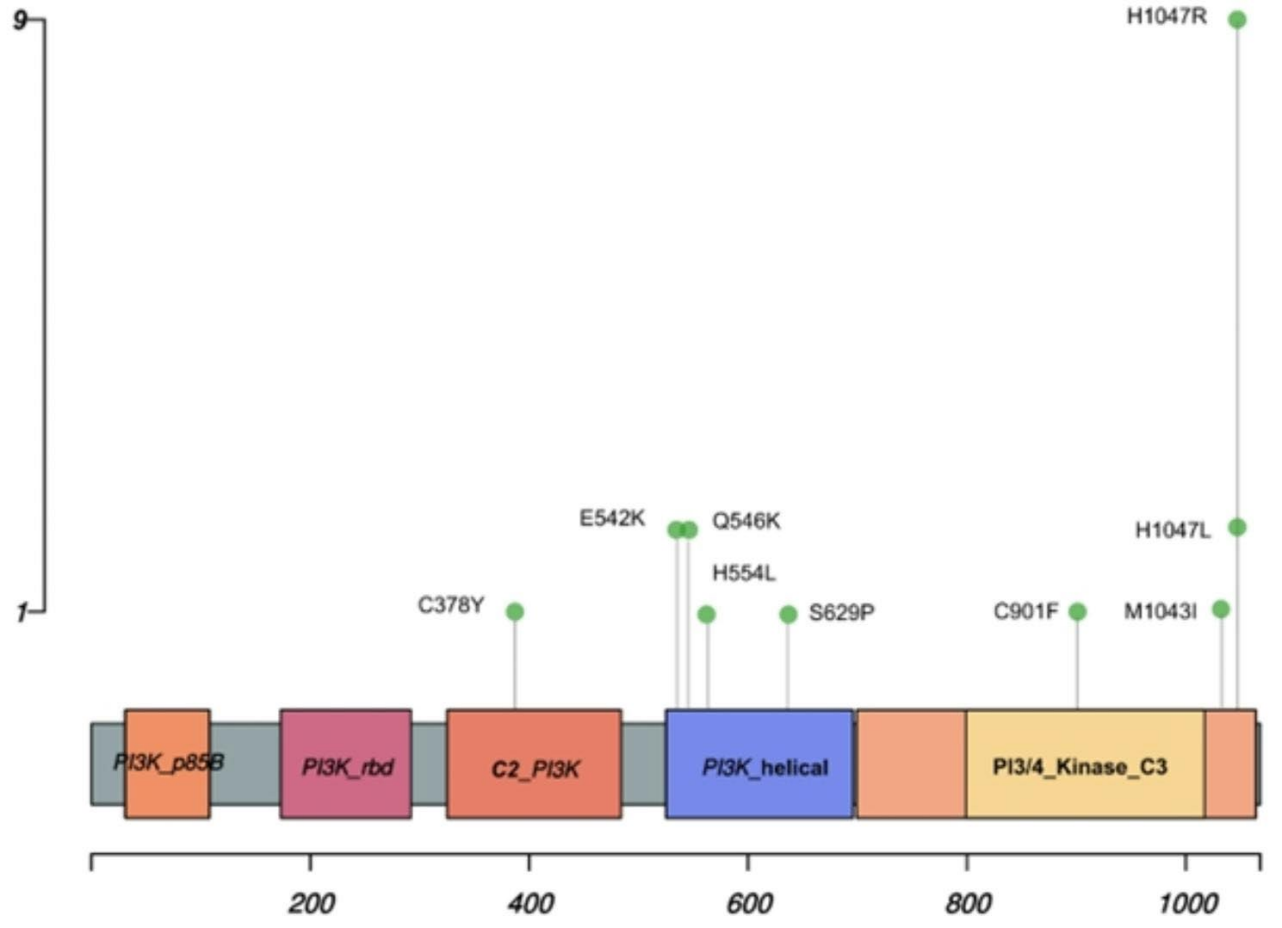
Genomic alteration of PIK3CA gene. Lollipop plot showing amino acid substitution at specific location. The height of vertical axis exhibits the number of cases while horizontal axis represents number of residues

The entire protein-coding regions of PIK3CA were sequenced using WES in each of the 45-breast cancer tissue as well as corresponding blood sample. A total of 17/45 tumor samples (37.8%) were found to carry a mutation in PIK3CA gene. No PIK3CA mutations were observed in the remaining 28 tumor samples (62.2%) (Fig. 1A) as well as blood samples. The standard variant calling program identified point mutation at exons 5, 9, 11, 18, and 20, and each mutation was supported by ≥ 5 reads. The visualization of reads having SNVs along with corresponding reference sequences was also done with an IGV in a web browser mode (Fig. S1). The mutations were usually base pair alterations, i.e., the substitution of a single nucleotide base with another base. Exons 9 and 20 had a maximum of 3 distinct SNVs, whereas the remaining exons (5, 11 and 18) carried only single point mutation (Fig. 1B). The distributions of these mutations are shown as exon 5 (p.C378Y; 1 case), exon 9 (p.E542K, p.Q546K, p.H554L; 5 cases), exon 11 (p.S629P; 1 case), exon 18 (p.C901F; 1 case) and exon 20 (p.M1043I, p.H1047R, p.H1047L; 12 cases) (Fig. 1C). Most of the cases carried a single mutation, while two cases had more than one mutation. The most frequent mutation was p.His1047Arg found in 9 cases (20%), followed by p.Glu542Lys (2 cases, 4.5%), p.Gln546Lys (2 cases, 4.5%) and p.His1047Leu (2 cases, 4.5%) whereas rest five mutations were found in one case (2.3%) each of the total mutation frequency (Fig. 2).

The 7 out of 9 SNVs (p.Cys378Lys, p.Glu542Lys, p.Gln546Lys, p.Cys901Phe, p.Met1043Ile, p.His1047Arg and p.His1047Leu) have been already reported in Catalogue of Somatic Mutations in Cancer (COSMIC) database (Table 2) and mainly found in breast cancer along with other cancer sites. At the same time, 2 (p.His554Leu and p.Ser629Pro) out of 9 SNVs are novel and have not been reported in COSMIC or any other genomic database till now. Though very low in frequency, these are probably the first times being characterized in our study. Additionally, molecular consequences of each variant were determined by MuPro, which showed that all variants scored higher than 0.5, indicating that these mutants are pathogenic in nature (Table S3). Out of 9, 3 SNVs, such as C378Y, H554L and S629P, also found with unknown pathogenicity.

**Table 2:** List of PIK3CA gene mutations and their annotation with COSMIC database

Sr. No.	gDNA Position	cDNA Position	Exon number	aaChange	Variant classification	Genomic Mutation ID	Tissue distribution (top 2)
1.	Chr3:g.179204576G > A	c.G1133A	Exon5	p.C378Y	Missense mutation	COSV55952006	Breast, Endometrium
2.	Chr3:g.179218294G > A	c.G1624A	Exon9	p.E542K	Missense mutation	COSV55873227	Breast, Large intestine
3.	Chr3:g.179,218,306 C > A	c.C1636A	Exon9	p.Q546K	Missense mutation	COSV55873527	Large intestine, Breast
4.	Chr3:g.179,218,331 A > T	c.A1661T	Exon9	p.H554L	Missense mutation	Not reported	
5.	Chr3:g.179219709T > C	c.T1885C	Exon11	p.S629P	Missense mutation	Not reported	
6.	Chr3:g.179230039G > T	c.G2702T	Exon18	p.C901F	Missense mutation	COSV55881149	Breast, Endometrium
7.	Chr3:g.179234286G > A	c.G3129A	Exon20	p.M1043I	Missense mutation	COSV55873376	Breast, Endometrium
8.	Chr3:g.173,234,297 A > G	c.A3140G	Exon20	p.H1047R	Missense mutation	COSV55873195	Breast, Large intestine
9.	Chr3:g.179,234,297 A > T	c.A3140T	Exon20	p.H1047L	Missense mutation	COSV55873401	Breast, Large intestine

Furthermore, their pathogenic status was also determined with the help of SNP prediction tools. It was found that 1 SNV (S629P) were deleterious in nature with a high cancer association rate whereas other C378Y and H554L are likely oncogenic [Table S4]. The most recurrent SNV (p.H1047R) was further validated through Sanger sequencing. However, the mutation was not detectable on electropherograms, possibly due to their very low variant frequencies (Fig. S2).

### ddPCR based validation of hotspot variants

**Fig. 3:**
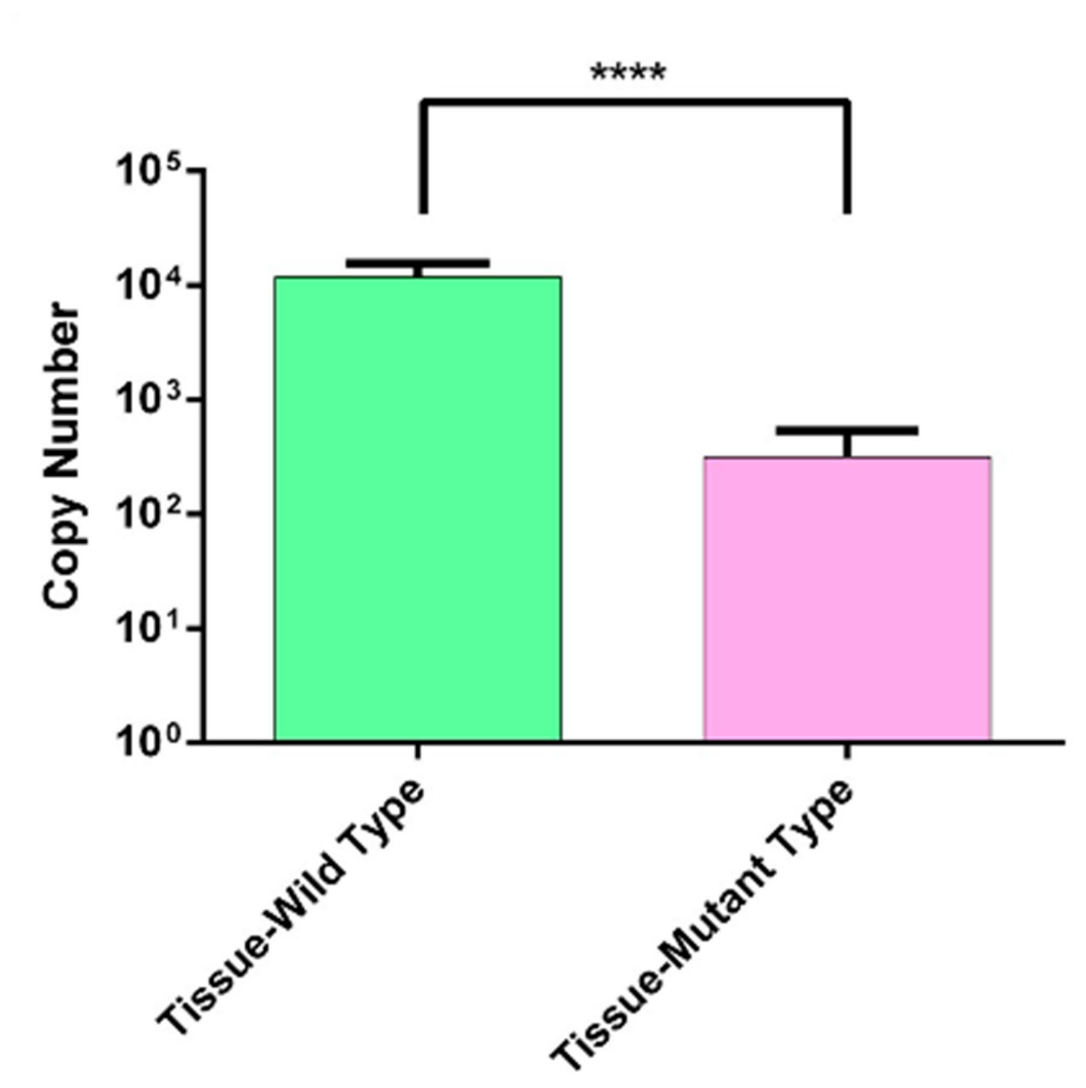
Validation of PIK3CA Mutation. Copy number distribution of PIK3CA wild Type and its mutant type (H1047R) in tissue samples. (* shows level of significance, p<0.0001).

To validate the hotspot variants (H1047R) with low variant allele frequency, we performed the digital probe-based PCR on 9 tumor cases DNA which was positive for H1047R on the IGV tool (Fig. S3). The mean value of copy number and the standard deviation were plotted using a graph pad prism, and a paired t-test was used for statistical analysis. A significant difference in the copy number of WT and MT was observed, with the percentages of mutants were found in the range of 0.36–4.72% (Fig. 3).

### Molecular dynamics simulation study

**Fig. 4:**
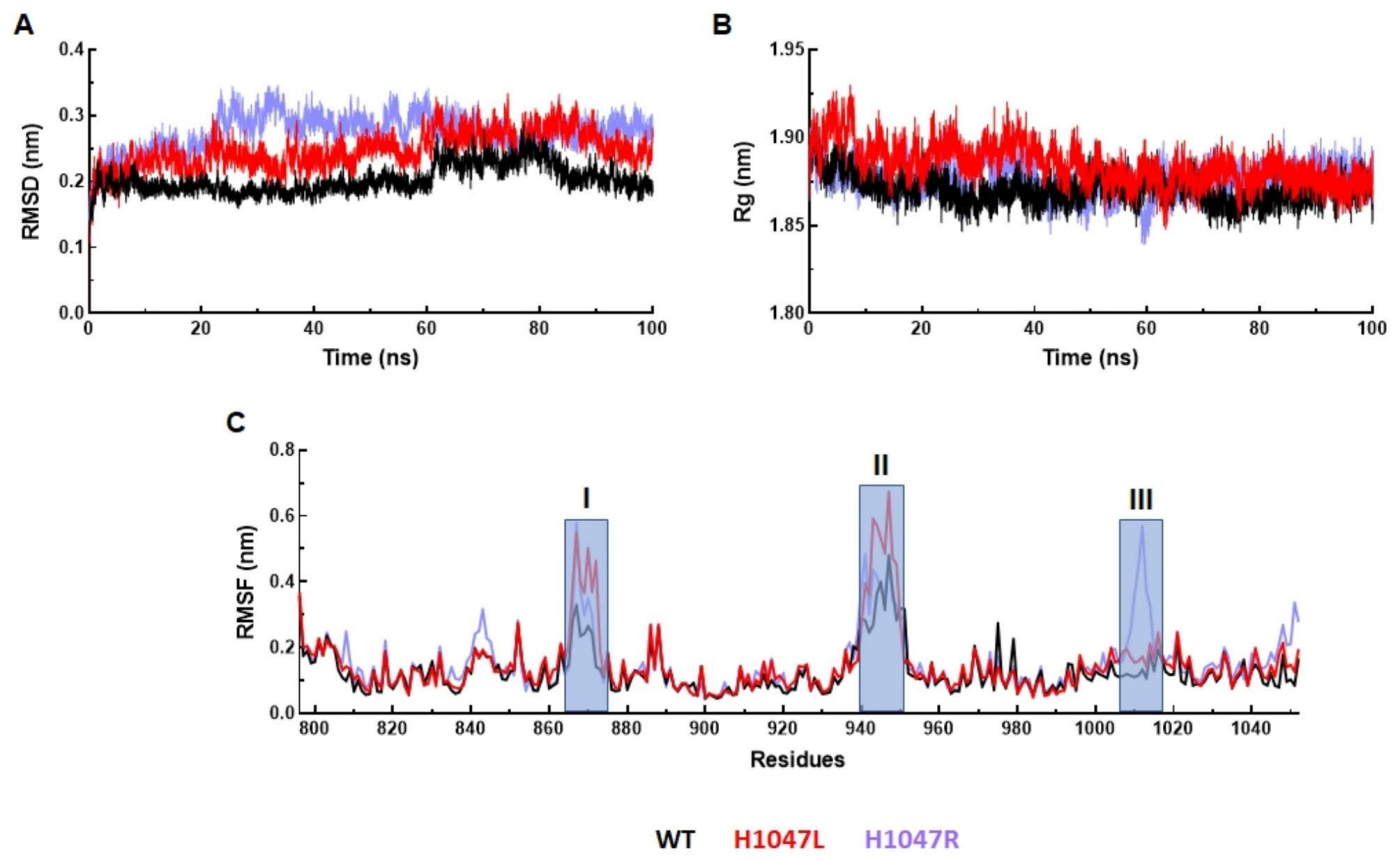
MD simulation profile of WT and MTs. (**A**) Root mean square deviation (RMSD measured in nanometer (nm) at function of time in nanosecond (ns), (**B**) Radius of gyration (Rg) measured in nm at function of time in ns, and (**C**) Root mean square fluctuation (RMSF) measured in nm at function of amino acid residues. WT, H1047L and H1047R MTs were labelled in black, red and blue lines, respectively. Highly fluctuated regions were highlighted in shaded rectangular boxes and indicated in regions I, II and III in RMSF graph

The tertiary structure of kinase domain of PIK3CA consists of 8 α-helices and 6 β-sheets (Fig. S4). Mutation in the kinase domain primarily affected the catalytic activity of PIK3CA. Out of 9 mutations, 3 were found in the kinase domain, and 2 of them positioned at hotspot 1047 in which Histidine was substituted by Leucine (H1047L) and Arginine (H1047R), which also contributed to chemotherapy resistance. We carried out MD simulation to study the effect of these mutations on the structures, conformation, and function of protein during the course of 100ns simulation time [41]. The differences between the backbones of a protein from its initial structural conformation to the final position was measured through root mean square deviation (RMSD) (Fig. S5). In case of WT, RMSD showed equilibrated behaviour with slight lifting at 80ns followed by stabilization till the end of simulation period of an average 0.20 nm RMSD (Fig. 4A). Similarly, RMSDs of both MTs (H1047L and H1047R) displayed steady behavior until the end of the simulation period with higher average values than WT (Fig. 4A). The average RMSDs of H1047L and H1047R MTs were about 0.27 and 0.24 nm, respectively. Further, the radius of gyration (Rg) was used to measure the compactness of protein structure during simulation period. Rg of WT and both MTs showed a similar pattern with average Rg of about 1.87, 1.88, and 1.87 nm for WT, H1047L, and H1047R, respectively (Fig. 4B). Hence, Rg of mutant proteins remains unaffected. The root mean square fluctuation (RMSF) of protein was calculated to determine the flexibility of the residues. WT and MT showed greatest fluctuations at residue numbers 865 to 875 and 940 to 950, in which H1047L showed maximum flexibility ≥ 0.6 nm RMSF, followed by H1047R and WT. While in the region from 1005 to 1015, H1047R showed the highest peak, which indicated highly mobile region (Fig. 4C).

**Fig. 5:**
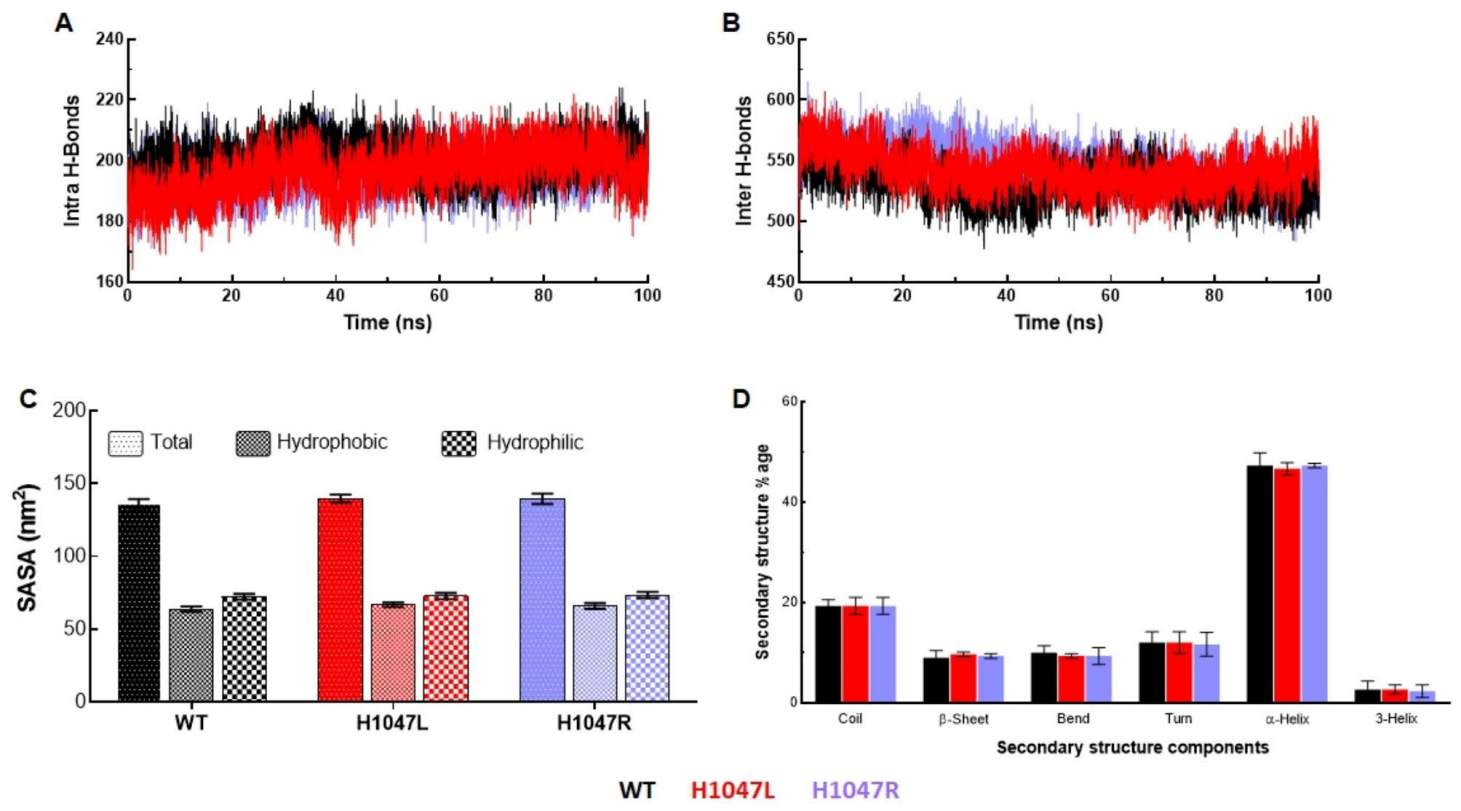
Structural properties analyses of WT and MTs. (**A**) Total number of protein-protein (intra) hydrogen bond formation in time of 100ns MD simulation, (**B**) Total number of protein-water (inter) hydrogen bond formation in time of 100ns MD simulation, (**C**) Solvent accessible surface area (SASA) analyses through measurement of hydrophobic and hydrophilic SASA, and (**D**) Secondary structures formation throughout the simulation period. WT, H1047L and H1047R MTs were labelled in black, red and blue lines, respectively

Different interactions within protein and protein-solvent contribute significantly in maintaining the integrity and stability of the protein. Intra and inter-molecular hydrogen bond (H-bond) formation were estimated from the 100ns MD simulated trajectories (Fig. 5A and B). An average number of intramolecular H-bond were calculated for WT, H1047L, and H1047R, which were around 200, 195, and 196, respectively. While in the case of intermolecular H-bond, H1047L (547) and H1047R (542) MTs showed a greater number of H-bond than WT (530). This indicated that the point mutations disrupted the overall intra and intermolecular H-bond to a greater extent as compared to WT. Overall stability and interaction surfaces of protein upon point mutations were examined by inspecting solvent-accessible surface area (SASA) (Fig. 5C and Fig S5). Higher values of total SASA were found in H1047L (139.55 nm^2^) and H1047R (139.56 nm^2^) as compared with WT (136.18 nm^2^) (Fig. 5C). The mean values of hydrophilic surface area were almost similar in WT (72.35) and H1047R MT (72.75), while H1047L MT (73.56) showed a slight gain in surface area. In the case of hydrophobic surface area, H1047L (66) and H1047R (66.81) MTs showed higher mean values as compared to WT (63.82) (Fig. 5C). The structure formation of WT and MTs composing various secondary structural moieties such as coil, β-sheet, bend, turn, α-helix, and 3-helix were studied throughout the simulation. H1047R, exhibited 20% coil and 10% bend, while WT and H1047L showed 21% and 19% coil contents with a similar percentage of bend (9%) (Fig. 5D). However, the similar fractions of β-sheet (10%) and 3-helix (2%) were found in all WT and MTs. Further, MTs showed low α-helix (48%) and turns (10%) contents as compared to WT (α-helix:50%; turns:11%) (Fig. 5D).

**Fig. 6:**
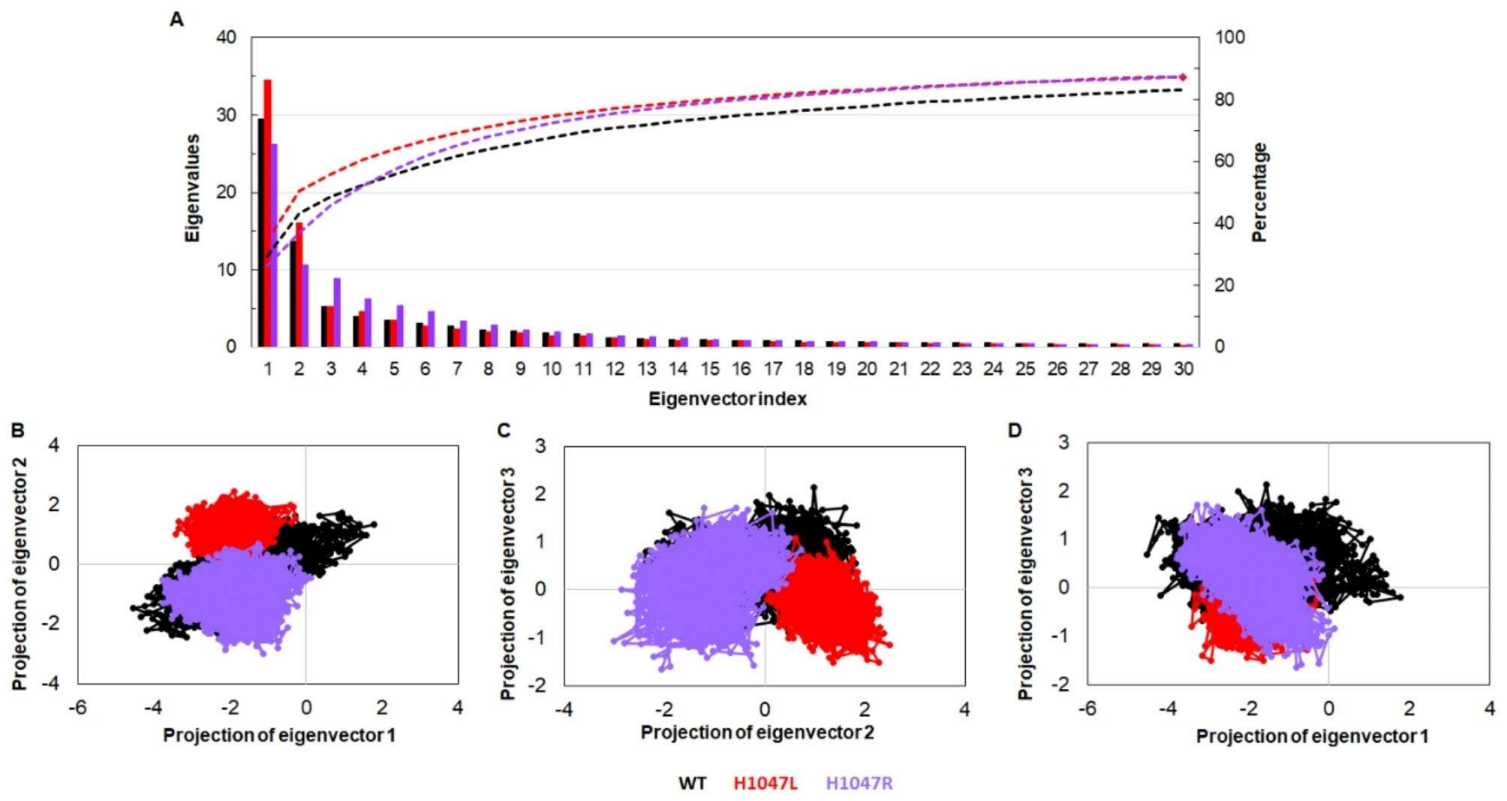
Collective mode of motion analyses of WT and MTs. (**A**) 2D plot of first 30 eigenvectors with corresponding eigenvalues and cumulative percentage of each eigenvector, (**B**) Projection of motions in phase space along the eigenvectors 1 versus 2, (**C**) Projection of motions in phase space along the eigenvectors 2 versus 3, and (**D**) Projection of motions in phase space along the eigenvectors 1 versus 3. WT, H1047L and H1047R MTs were labelled in black, red and blue lines, respectively

We performed essential dynamics by utilizing the stable trajectory of respective systems to inspect the collective motions and conformational changes induced upon mutations [42]. The first 30 eigenvectors or principal component (PC) exhibited 80% of motions, which had eigenvalues > 1 nm^2^ in WT, and MTs were carried. Out of this, the first 3 PCs largely contributed with an account of 48.5% (WT), 45.8% (H1047L), and 55.9% (H1047R) (Fig. 6A). Scatter plots were generated from the principal component analysis (PCA) of WT and MTs were shown in Fig. 6B-D. It showed the overall motions of the systems (WT, H1047L, and H1047R) with respect to their corresponding PCs. WT covers a maximum area in space resembling dominant motions, while MTs show restricted movement and were confined within smaller subspaces. Additionally, for a better understanding of the variations in the collective motion of each system, the dynamic nature of WT and MTs were examined by successively superimposing 30 frames from the first PC for each system (Fig. S6). Furthermore, the motions of the protein in particular directions were examined through porcupine plot, and it was found that motions were mainly restricted to C-terminal (Fig. S6). No such movements were observed in the N-terminal regions of WT and H1047L MT, while the C-terminal of WT and H1047R showed minor motions. The kinase domain of WT shows significant motions, which were not observed in both the MT protein structures.

## Discussion

The prognosis and chemotherapeutic response of tumor depend upon multiple variables, including histopathological grade, staging, groups, HR and Her2 status. These factors are taken into consideration by researchers and clinicians to classify breast cancer. On that basis, suitable diagnosis and treatment are offered to cases to achieve the best prognosis. Generally, the development of cancers is driven by mutations in the DNA, which triggers molecular abnormalities in the cell cycle. Depending upon the function of mutated gene, this mutation may be pathognomonic or non-pathognomonic, and it may also influence the prognosis. These alterations are either activated through the activation of oncogenes or by silencing the tumor suppressor genes. These are involved in various cell signalling, apoptosis, and cell differentiation pathways. PI3K is one such canonical pathway that is involved in signal transduction through receptor tyrosine kinases, or G-protein coupled receptors, and is associated with cell growth, motility, survival, proliferation, and apoptosis in response to external stimuli [43, 44].

Activation of the PI3K pathway is frequently observed in the pathogenies of several solid cancer, including BC, in which PIK3CA mutation was predominantly observed [45, 46]. It is located on the long arm (q) of chromosome 3 and composed of various domains such as p85 binding, receptor binding, membrane binding (C2), helical and kinase (C3) domains as shown in Fig. 2. Activation of catalytic subunit require its interaction with the phosphotyrosine residues of activated growth factor receptors [47]. As a result, phosphatidylinositol-3,4,5-trisphosphate (PIP3) is generated from the membrane lipid phosphatidylinositol-4,5-bisphosphate (PIP2) and, in turn, triggers AKT. Consequently, it encourages various down-streaming biological processes [48].

Previous studies suggested that 18–40% of BC cases harbor PIK3CA mutations compared to approximately 38% in our study [49–51]. Moreover, in several other cancer related studies, the frequency of PIK3CA was reported as high in endometrial (36%), head/neck squamous cell carcinoma (33%), and colon cancer (32%). On the contrary, studies on oral squamous cell carcinoma, and lung have reported low incidence as low as 7% and 4%, respectively [52, 53]. This discrepancy of the mutation incidence pattern is possible outcome of various factors such as biologically different squamous epithelial origin, exposure to tobacco and environmental pollutants.

In our study, breast cancer has been divided into four categories depending on the hormone receptor status. Hence, the different subtypes of BC, the prevalence of PIK3CA mutation varies. In our cases, we found higher incidence of PIK3CA mutation in estrogen-positive subtypes, compared to Her2 enriched and basal-like BC, as similar with other previous studies [54]. In the present study, the expression of the PIK3CA gene was upregulated in control adjacent tissue in comparison with breast carcinoma tissue. Similar results were also found in the studies performed by Kolodziej et al. and Cizkova et al. [55, 56]. The lower expression was observed, possibly due to the correlation of expression with the normal group.

The hotspot PIK3CA mutation mainly lies in the helical and kinase domains [52]. The helical domain plays an important role in the allosteric regulation of the kinase domain. It receives a signal from the regulatory subunit (p85) via the SH2 domain and further control the activity of kinase region. Upon mutation, it allows escaping from the inhibitory effect of p85, which makes the hyperactivation of kinase domain [57]. While exon 20 lies in the kinase region, and its mutation helps to achieve a gain of function as well as alter its transforming capacity [58]. Helical domain mutations such as E542K and E545K and kinase domain mutations such as M1043I and H1047R are known to increase lipid kinase activity in many cancers, including BC. Our study showed no evidence of E545K mutation but Q546K was found in 2(4.5%) cases. The substitution of glutamine with lysine residue at 546th position introduces a positive charge that forms hydrogen bond with other negatively charged atoms which alters the binding sites and recruits other molecules to the membrane [59]. In comparison, the other non-synonymous mutations such as C378Y, H554L, S629P, C901F, and H1047L have different hydrophobicity. In C378Y, hydrophobic interaction will be lost either within the core or on the protein’s surface. At the same time, the hydrophobic interaction increased in the case of H554L, S629P, C901F, and H1047L.

Further, we used molecular dynamics simulation to gain insight into structural consequences of the substitution of His1047 with Arg or Leu. The variations obtained through the simulation process can be used to determine the protein’s stability in relation to its conformation. MD simulation results suggested that the WT maintained overall stability while the MTs displayed more fluctuations. The net dimension of protein was analyzed by performing Rg. The similar values of Rg across WT and MTs revealed no such major transition in compactness and globularity. To identify the dynamics of residues, the RMSF of WT and MTs were determined. Highlighted regions between 865 and 875, 940–950, and 1005–1015 showed higher flexibilities of backbone residues in MTs than WT. Thus, the mutant increased the flexibility of PIK3CA and made tertiary structure (3D) unstable. The instabilities of mutant proteins in the 3D conformational space were supported with RMSD as well as RMSF analyses.

**Fig. 7:**
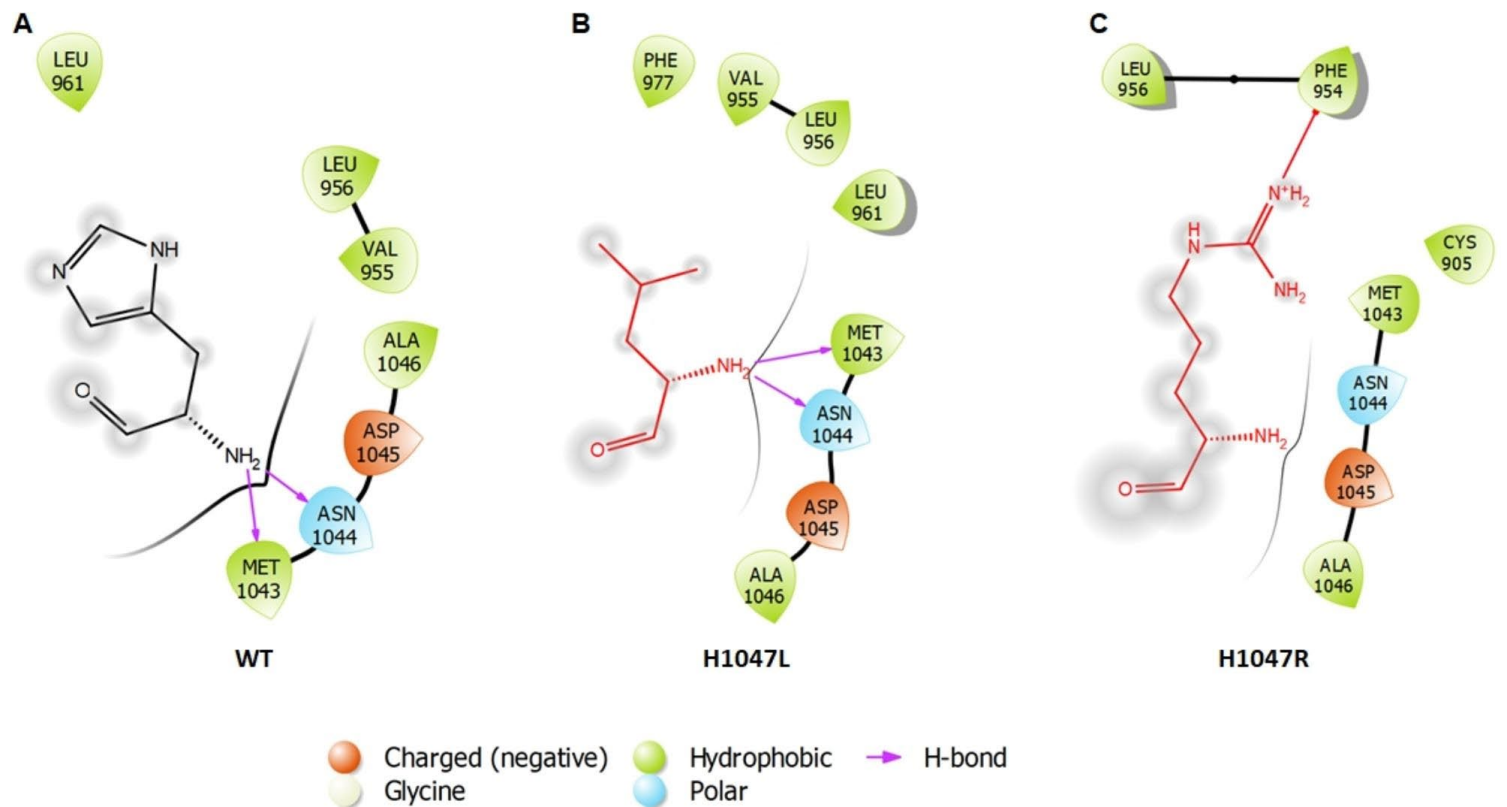
Residue Interaction Analysis. 2D interaction map of (**A**) H1047 wild type residue (**B**) H1047L mutant residue (**C**) H1047R mutant residue

The tertiary structural stabilities of proteins were studied by analysing their H-bond pattern. We studied 2 different types of H-bonds- intra H-bonds (establish in the protein molecule among the adjacent residues) and inter H-bonds (formed between the protein and solvent molecules or ligand). H-bond formation provides a clear understanding about the change in structural conformation of the protein as well as their intercommunication. According to H-bond studies, more intra-H-bonds were observed in WT, while more inter-H-bonds were found in MTs. This suggested that MT proteins had developed flexible and distorted structures. The dynamics of protein play a vital role in ligand or substrate binding and conformational adaptation, which can be studied through phase space behaviour using essential dynamics approach. The majority of the protein dynamic behaviour were found in first 3 PCs, and the spectrum of corresponding eigenvector indicated about the motion of a protein in space. It was observed that MTs showed restricted and confined motions as compared to WT, thus indicating that protein had unstable conformation upon the mutations. The network biology approach was used to identify the interacting residues along with their types of bonds within their surrounding environment. Each node in a network represents residue, whereas edges depict various forms of interactions between them. From the 2D interaction maps analyses, we found that in WT, His at 1047 position formed 2 hydrogen bonds with Met 1043 and Asn 1044. A similar pattern of H bonding was observed when His 1047 was replaced with Leu 1047. On the other hand, the substitution of His 1047 to Arg 1047 abolished the hydrogen bond between the Met 1043 and Asn 1044 (Fig. 7).

In the present clinical scenario, PIK3CA is examined as an important target therapy in BC management. PIK3CA mutations are reliable predictors of how cases will respond to specific PI3K inhibitors [60]. About 30% estrogen-positive (Er+) BC harbors mutation in the catalytic domain of PIK3CA [61]. Usually, BC cases diagnosed with Er + subgroups underwent endocrine therapy (ET) treatment and demonstrated to be the gold standard because of its significant outcome. However, over time being, a significant percentage of BC cases develop ET resistance by virtue of its accumulation of H1047R mutation in the catalytic domain of PI3K [62]. Apart from all its concerns, routine PIK3CA genotyping is still lacking in current practice. In addition, other PIK3CA mutation could be validate by experimental studies to understand their role in prognosis as well as response towards chemotherapy.

## Conclusions

In lower-middle-income or developing countries, molecular testing for genetic and genomic variants has become crucial for breast cancer management. The current study provides a comprehensive view of PIK3CA mutation associated with Indian breast cancer cases and identified novel as well as reported SNVs. However, our study has some drawback, such as cohort with small number of BC cases, which limits to draw a concrete conclusion. Therefore, current finding must be verified in large cohorts participating in randomised clinical trials.

### Electronic supplementary material

Below is the link to the electronic supplementary material.

Supplementary Material 1

## Data Availability

The datasets generated and/or analysed during the current study are available in the NCBI repository having BioProject number PRJNA935139.

## List of abbreviations

BC: Breast cancer
BQSR: Base quality score recalibration
BWA: Burrows-Wheeler Aligner
COSMIC: Catalogue of Somatic Mutations in Cancer
ddPCR: Droplet digital PCR
ET: Endocrine therapy
gDNA: Genomics DNA
GQ: Genotype quality score
IGV: Integrative Genomics Viewer
LINCS: Linear constraint solver
MD: Molecular dynamics
Particle Mesh Ewald: PME
PCs: Principal components
Phosphate buffer saline: PBS
PI3K: Phosphoinositide 3-kinase
PIP2: Phosphatidylinositol-4,5-bisphosphate
PIP3: Phosphatidylinositol-3,4,5-trisphosphate
Protein Structure Analysis: ProSA
RED: Rare event detection
Rg: Radius of gyration
RMSDs: Root mean square deviations
RMSF: Root mean square fluctuation
SNVs: Single nucleotide variants
