# Supplementary material for: Whole exome sequencing identifies novel variants of PIK3CA and validation of hotspot mutation by droplet digital PCR in breast cancer among Indian population
Source: Cancer Cell Int. 2023 Oct 11;23:236. doi: 10.1186/s12935-023-03075-6 (PMC10568783; doi:10.1186/s12935-023-03075-6)
Supplement: Supplementary file 1 — Supplementary Material 1 [file 12935_2023_3075_MOESM1_ESM.pdf]

Supplementary materials

Whole exome sequencing identifies novel variants of PIK3CA and validation of hotspot mutation by droplet digital PCR in Breast cancer among Indian Population.

Rahul Kumar[1], Rakesh Kumar[1], Harsh Goel[1], Sonu Kumar[2], Somorjit Singh Ningombam[1], Imran Haider[1], Usha Agrawal[3], SVS Deo[1], Ajay Gogia[1], Atul Batra[1], Ashok Sharma[4], Sandeep Mathur[5], Amar Ranjan[1], Anita Chopra[1], Showket Hussain[6], Pranay Tanwar[1*]

[1]Dr B. R. A.-Institute Rotary Cancer Hospital, All India Institute of Medical Sciences, New Delhi, India

[2]Department of Gastroenterology & HNU, All India Institute of Medical Sciences, New Delhi, India

[3]National Institute of Pathology, New Delhi, India

[4]Department of Biochemistry, All India Institute of Medical Sciences, New Delhi, India

[5]Department of Pathology, All India Institute of Medical Sciences, New Delhi, India

[6]Division of Molecular Oncology, National Institute of Cancer Prevention and Research, Noida, India

***Correspondence**: pranaytanwar@aiims.edu

Running title–Mutational landscape of PIK3CA in breast cancer.

Total supplementary figures : 6

Total supplementary tables : 5

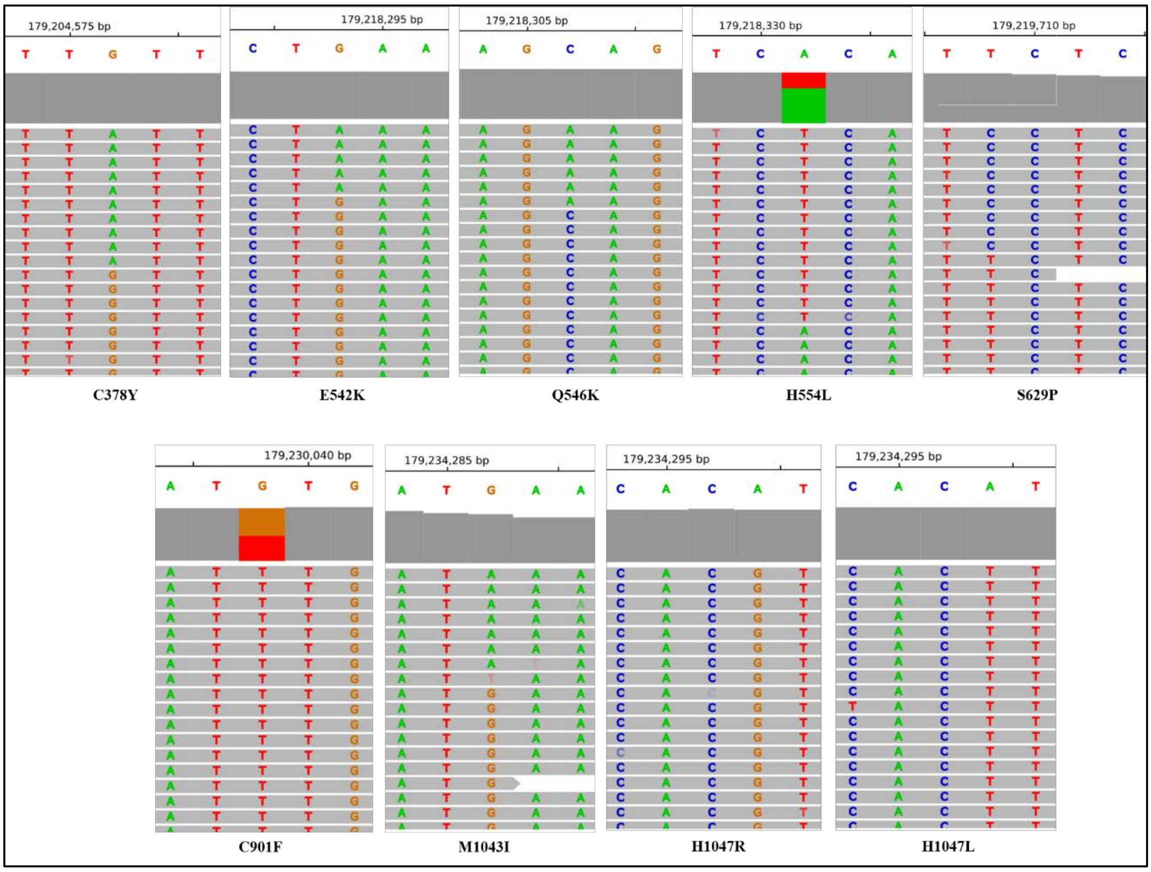

Fig. S1 Visualization of integrated genomic mutant reads (A) C378Y, (B) E542K, (C) Q546K, (D) H554L, (E) S629P, (F) C901F, (G) H1047R, (H) H1047R and (I) M1043I were accomplished by Integrative genomic viewer **(**IGV).

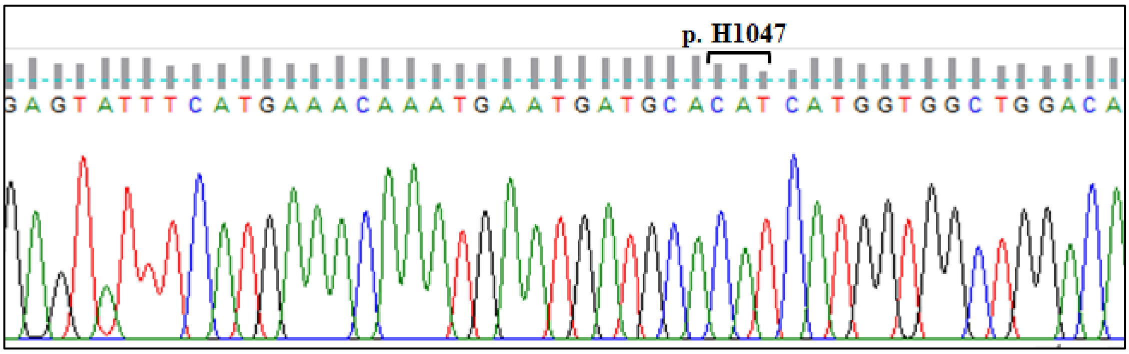

Fig. S2 Detection of H1047R by performing Sanger sequencing. H1047R missense mutation was not detected in breast cancer cases when compared with the reference genome due to low variant frequency**.**

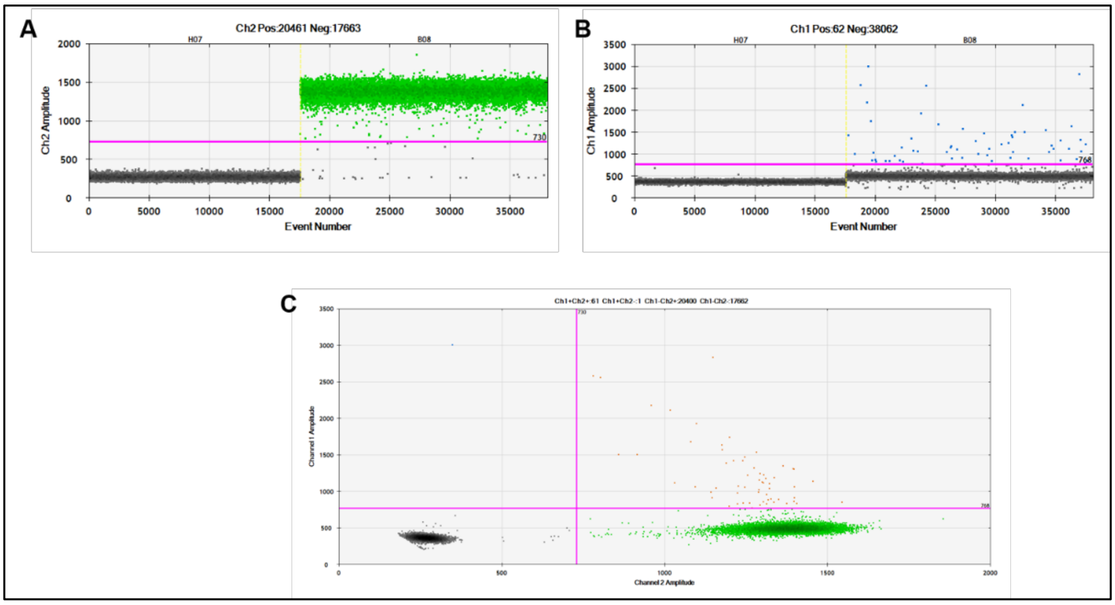

Fig. S3 Single plex ddPCR assay for identification of PIK3CA wild type and mutant. Tumor DNA of BC (A) mutant probe labelled with FAM fluorophore, (B) wild probe labelled with HEX fluorophore and (C) 2D cluster plot of rare event detection in which channel 1 mutant probe plotted against channel 2 wild probe.

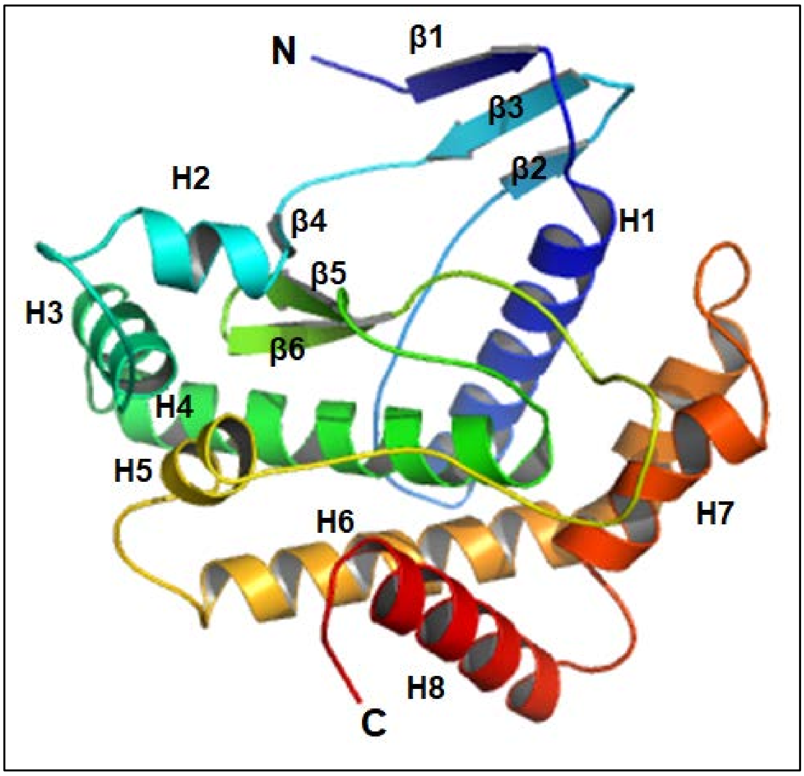

Fig. S4 Tertiary structure of kinase domain of PIK3. Both N and C-terminals are indicated and helix and sheet numbers are labelled as H and β symbols. Structure is displayed in publication cartoon mode and rendered in PyMOL graphical system.

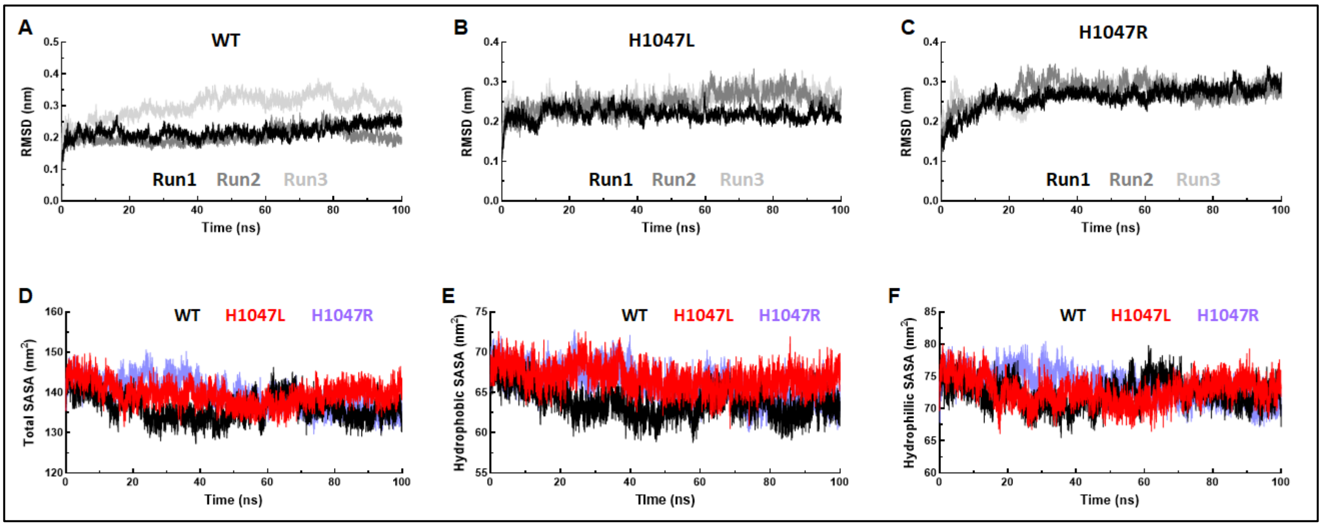

Fig. S5 Root mean square deviation of (A) WT, (B) H1047L and (C) H1047R, Solvent accessible surface area with (D) Total SASA, (E) Hydrophobic SASA and (F) Hydrophilic SASA. MD simulation is performed in triplicates and WT, H1047L and H1047R MTs were labelled in black, red and blue lines, respectively.

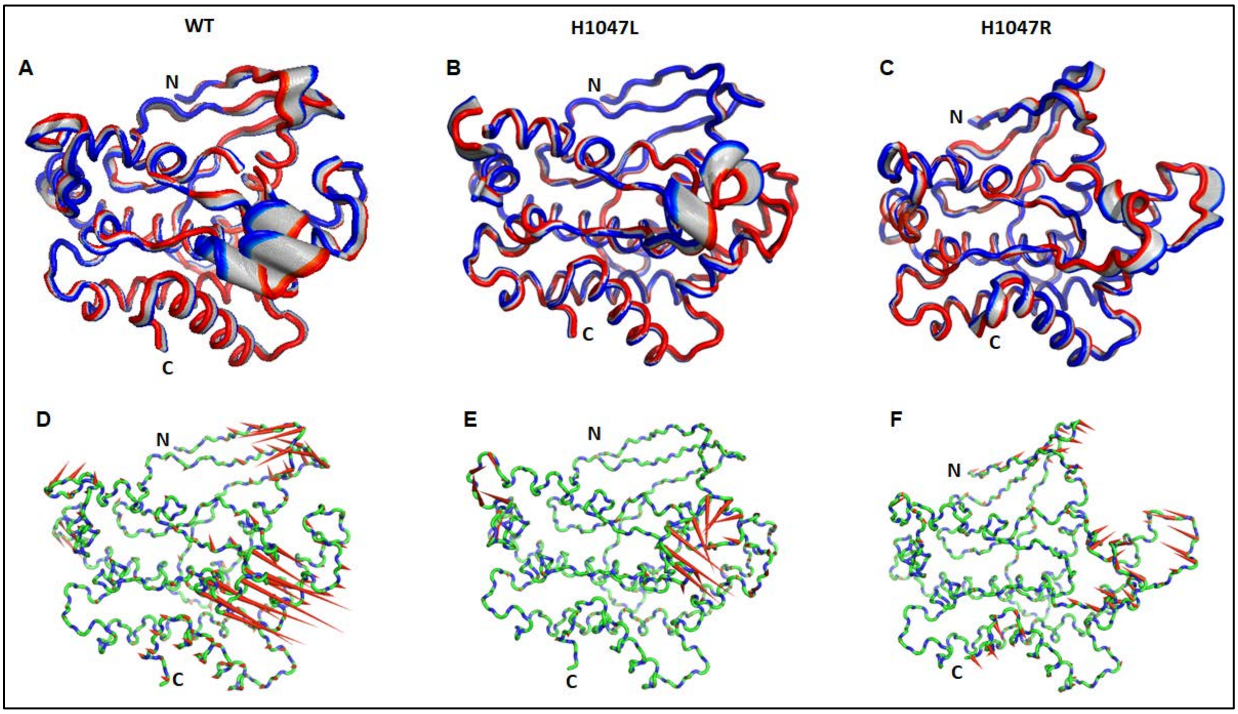

Fig. S6 Collective mode of motions of(A) WT, (B) H1047L and (C) H1047R (upper panel) with corresponding porcupine structure in lower panel (D-F). All structures are generated in PyMOL graphical system. Red and blue regions in upper panel structures indicated minimum and maximum motions, respectively. In porcupine structure, the cone and length indicated the direction and amplitude of motions. Both N- and C-terminals are indicated in all 3D structures.

Table S1. Primers for PCR and qRT-PCR

Gene	Forward Primer	Reverse Primer
PIK3CA	CTGAGCAAGAGGCTTTGGAGTA	CAATCGGTCTTTGCCTGCTGA

Table S2. Assay ID for ddPCR

	DDPCR assay ID	COSMIC ID
PIK3CA WT for p.H1047R	dHsaMDW5225715853	COSV55873195
PIK3CA p.H1047R	dHsaMDM5225715851	COSV55873195

Table S3. Functional annotation of PIK3CA variants

Variants	MutPred2 score	Molecular consequences	Probability	P-value
C378Y	0.893	Altered Ordered interface	0.36	3.3e-03
		Gain of Strand	0.30	2.3e-03
		Altered Transmembrane protein	0.11	0.03
E542K	0.729	Loss of Loop	0.27	0.03
Q546K	0.833	-	-	-
H554L	0.830	-	-	-
S629P	0.818	Loss of Helix	0.27	0.04
C901F	0.971	Altered Metal binding	0.40	6.5e-03
		Gain of Strand	0.27	0.03
		Altered Transmembrane protein	0.19	6.3e-03
		Loss of Catalytic site	0.18	0.02
M1043I	0.815	Altered Metal binding	0.21	0.03
		Altered Transmembrane protein	0.13	0.02
H1047R	0.831	Altered Ordered interface	0.31	0.02
		Altered Metal binding	0.28	5.3e-03
		Altered DNA binding	0.23	9.5e-03
		Altered Transmembrane protein	0.21	4.0e-03
H1047L	0.870	Altered Metal binding	0.28	5.8e-03
		Altered Transmembrane protein	0.23	2.7e-03
		Altered DNA binding	0.18	0.03
Variants	MutPred2 score	Molecular consequences	Probability	P-value
C378Y	0.893	Altered Ordered interface	0.36	3.3e-03
		Gain of Strand	0.30	2.3e-03
		Altered Transmembrane protein	0.11	0.03
E542K	0.729	Loss of Loop	0.27	0.03
Q546K	0.833	-	-	-
H554L	0.830	-	-	-
S629P	0.818	Loss of Helix	0.27	0.04
C901F	0.971	Altered Metal binding	0.40	6.5e-03
		Gain of Strand	0.27	0.03

		Altered Transmembrane protein	0.19	6.3e-03
		Loss of Catalytic site	0.18	0.02
M1043I	0.815	Altered Metal binding	0.21	0.03
		Altered Transmembrane protein	0.13	0.02
H1047R	0.831	Altered Ordered interface	0.31	0.02
		Altered Metal binding	0.28	5.3e-03
		Altered DNA binding	0.23	9.5e-03
		Altered Transmembrane protein	0.21	4.0e-03
H1047L	0.870	Altered Metal binding	0.28	5.8e-03
		Altered Transmembrane protein	0.23	2.7e-03
		Altered DNA binding	0.18	0.03

Table S4. Pathogenicity prediction of variant of unknown significance

**Variants**	**Predict SNP**	**FATHMM**	**SNPs&GO**	**Mutation assessor**	**Mutation Taster**
C378Y	Neutral	Cancer	Disease	Medium	Disease
H554L	Neutral	Cancer	Neutral	Neutral	Disease
S629P	Deleterious	Cancer	Disease	Medium	Disease

Table S5. Cosine content values for first 3 PCs

		**1-25**	**26-50**	**51-75**	**76-100**
**WT**	PC1	0.0452676	0.0360703	0.41195	0.210914
	PC2	0.174698	0.138471	0.573618	0.430119
	PC3	0.342793	0.0150176	0.00860085	0.0302362
**H1047R**	PC1	0.0664025	0.518629	0.00544277	0.0145975
	PC2	0.441048	0.0448567	0.493826	1.14E-05
	PC3	0.00382353	0.315541	0.0803878	0.025208
**H1047L**	PC1	0.0354101	0.303033	0.0458024	0.0312635
	PC2	0.0201197	1.73E-01	0.223455	0.0660624
	PC3	0.298651	0.613113	0.593642	0.384831
